# A Tried and Tested Waterproof Draping Method for Major Ear Surgery

**DOI:** 10.7759/cureus.32945

**Published:** 2022-12-26

**Authors:** Craig Hickson, Sheikh Muktadir Bin Momin, Kusum Asnani, Codruta Neumann

**Affiliations:** 1 Otolaryngology, William Harvey Hospital, East Kent Hospitals University NHS (National Health Service) Foundation Trust, Ashford, GBR

**Keywords:** continuing medical education/graduate medical education/undergraduate medical education, draping, external ear, neuro-otology, otology

## Abstract

Preparing and maintaining a clean operative field is the standard of care in all surgical fields globally. Major ear surgery has its own challenges such as the long surgical procedure time and the tricky local anatomical landscape. A waterproof method of draping for major ear surgery is described in this technical report. This method allows for the collection of irrigation fluid in a reservoir while maintaining continued isolation of the operative field during surgery. We discuss the advantages of using a 3M Steri-Drape^TM^ Aperture Pouch Drape to square the surgical site and create a pouch dedicated to irrigation fluid. Following that, running locking stitches are performed for further reinforcement of the adhesion to the skin, often done in longer procedures. We have identified a technique to ensure better draping. In over 150 cases draped in this method, we have not witnessed drape edge lift, water ingress, or skin avulsion/injury.

## Introduction

Antiseptic cleaning of the skin and isolation of the surgical site from areas that remain 'dirty' is the standard of care in all surgical fields globally [1]. The purpose of this practice is to prepare and maintain a clean operative field to prevent wound contamination and consequent post-operative infection. Major ear surgery is no different from any other surgery in this requirement; however, its often prolonged duration and local anatomical landscape, including the shoulder, neck, and head, can make maintaining an antiseptic surgical field problematic.

Standard surgical draping of the ear for mastoid procedures requires larger post-auricular access than that provided by standard fenestrated drapes commonly employed for minor ear procedures such as myringotomy. Consequently, surgeons are often left to drape the surgical site by 'squaring off' using standard rectangular drapes or head drapes in combination. It is the author’s experience having witnessed major ear surgery in over 10 different hospitals by 10 experienced otologists that such draping often leads to peeling or lifting of the adhesive edges of the drapes such that there is communication between the surgical field and the areas under the drapes. Even when sutured in place, these gaps develop because of forces applied to the drapes by the operating surgeon and equipment, as well as water ingress under the adhesive edges from irrigation fluid.

We describe a waterproof method of draping for major ear surgery which ensures continued isolation of the operative field throughout major ear surgery and allows the collection of irrigation fluid in a reservoir. This method of draping is compatible with recently developed COVID-19 isolation chambers and drapes such as the COVID-19 Airway Management Isolation Chamber (CAMIC) and drape 'tent' recently described in the literature [2,3]. We received approval from our Institutional Research and Innovation Department (approval number: 2021/GAP/21) to involve human participants and procedures in our study.

## Technical report

**Figure 1:**
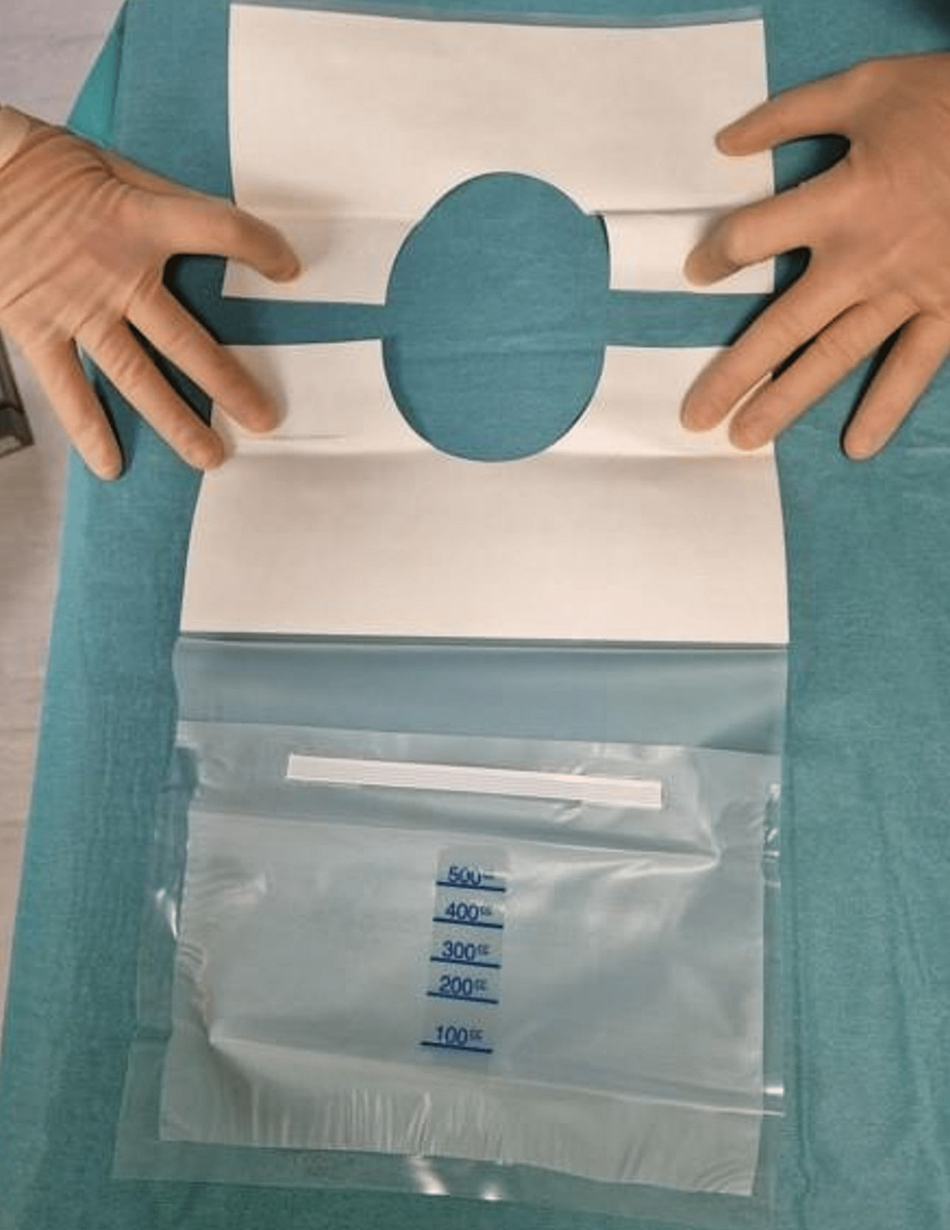
3M Steri-DrapeTM Aperture Pouch Drape.

**Figure 2:**
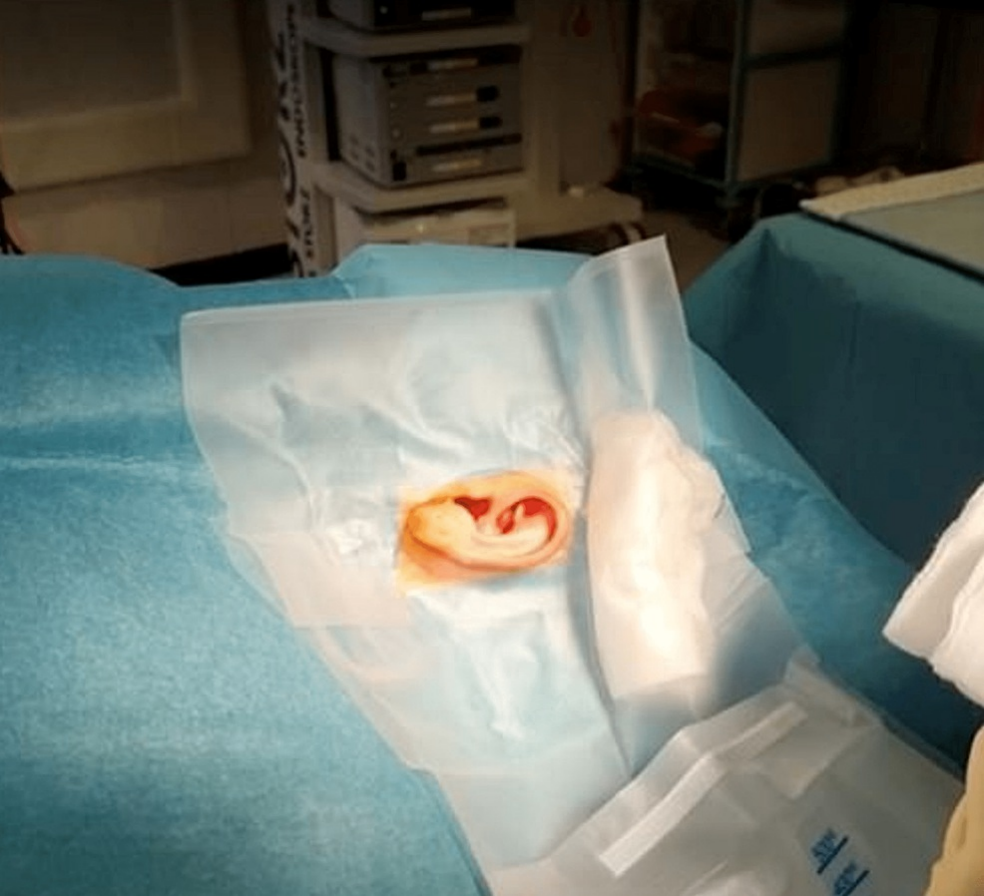
Steri-DrapeTM positioned overlapping the leading edges of the squared-off drapes and adhering them together.

Following preparation of the surgical field using an antiseptic of the surgeon’s choice, and allowing for drying time, the surgical site is 'squared off' using standard rectangular surgical drapes with adhesive edges. Following this, a 3M Steri-Drape^TM^ Aperture Pouch Drape (as shown in Figure 1) is applied with the adhesive overlapping the squared-off drapes (Figure 2).

**Figure 3:**
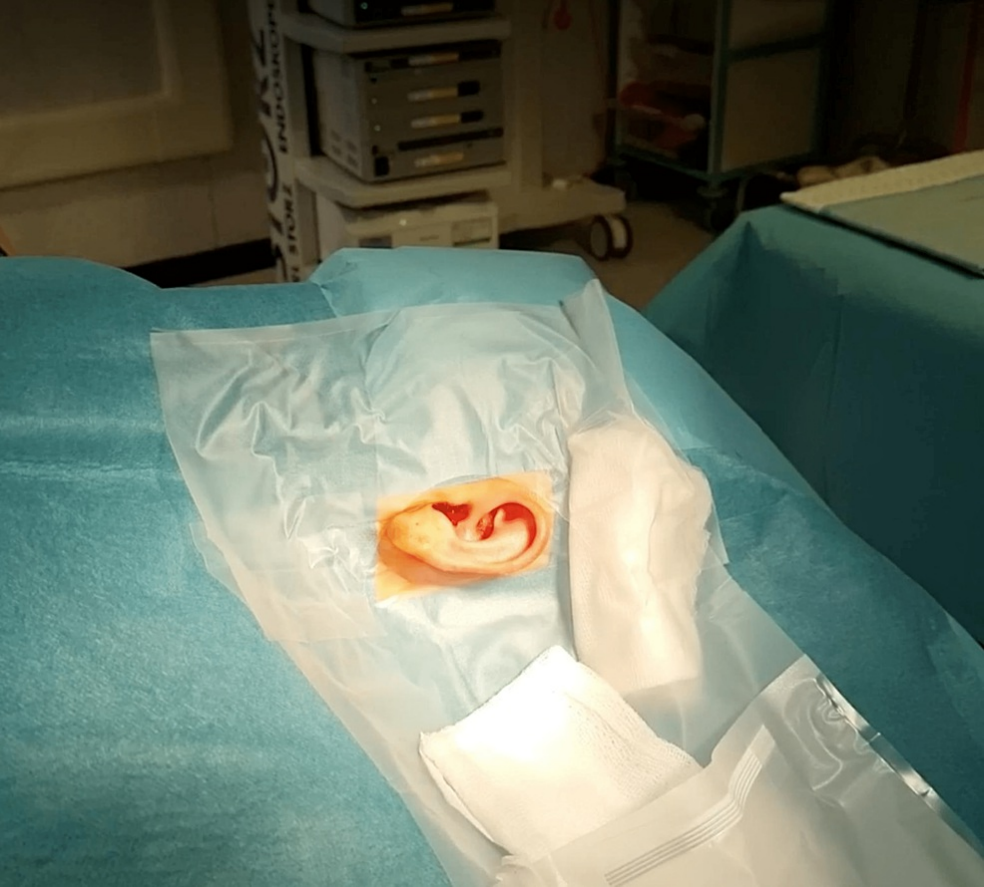
Folds and swabs have been used to guide irrigation fluid into the drape pouch.

The Steri-Drape^TM^ provides three functions: 1. It sticks all four of the standard drapes together, making it impossible for one of them to loosen on its own. 2. There is considerable overlap of the adhesive Steri-Drape^TM^ onto the skin reinforcing the adhesion to the patient’s skin. 3. By placing folds into the Steri-Drape^TM^ and swabs under it, the irrigation fluid is guided into the pouch where it can be evacuated, keeping the drapes and surgeon’s legs dry (Figure 3).

**Figure 4:**
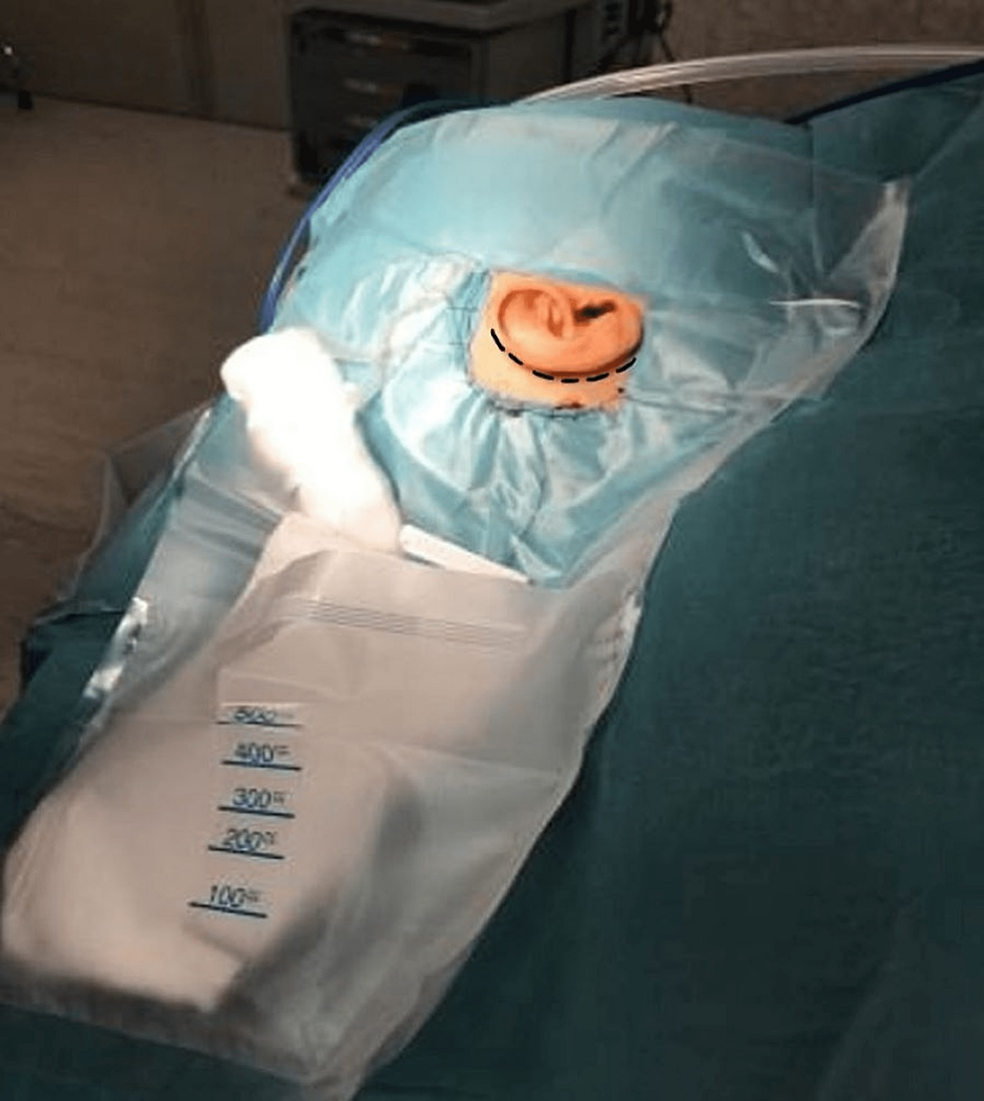
Running locking stitches of the drapes to the patient’s skin provides extra security against the lifting of the adhesive edges of the drapes. The post-auricular incision is drawn on the figure, it is a few centimetres away from the drape.

Further reinforcement of the adhesion to the skin can be ensured by suturing the Steri-Drape^TM^ to the patient’s skin using a running locking stitch (2/0 silk) at the surgeon’s discretion (Figure 4). Our draping technique is shown in the attached video (Video 1). In our experience of over 150 such cases, we have not witnessed drape edge lift, water ingress, or skin avulsion/injury consequent with our method of draping.

**Video 1:** Demonstration of a tried and tested waterproof draping method for major ear surgery.

## Discussion

Slippage or movement of surgical drapes during surgery is a well-recognised phenomenon. With the advent of adhesive drapes or drapes with adhesive edges, better isolation of the surgical field is achieved when compared to drapes that are secured in place with towel clips. Even so, forces applied to the drapes and the effect of water penetrating the drapes often result in suboptimal adhesion and gaps forming. This method of ear draping excludes hair and its associated infection risk when trapped in the drill or when fragments of hair enter the mastoid or middle ear.

The combination of the drapes and using a running locking stitch (2/0 silk) is often done in longer procedures when the adhesive tapes on the drapes loosen due to sweat and degree of traction from the surgical equipment. Given the significantly adhesive nature of the Steri-Drape^TM^, one must be mindful of the potential for skin avulsion injuries when using any adhesive drapes. While injuries from adhesive drapes have been reported, we have not experienced any such injury to date [4]. With careful removal and recognition of those patients where such injury may occur, such injury should be avoidable.

Although an adhesive drape applied over the entirety of the squared-off surgical field would provide similar fixation and waterproofing, the ear itself prevents such drapes from being laid flat against the skin. In addition, while waterproofing the field, such a drape would not provide the reservoir that our method provides. We find such a reservoir invaluable in keeping a dry field and dry knees. Keeping the patient’s head dry minimises the risk of skin pressure sores during lengthy mastoid procedures.

## Conclusions

The technique described above provides a robust, waterproof, fluid-collecting barrier that is well-suited for major ear and mastoid procedures. It is unique in enabling the creation of a pouch for irrigation fluid and ensuring safe adhesion to the patient’s skin during the procedure. This technique helps to overcome issues that would have been otherwise experienced in preparing the surgical field for major ear surgery.
